# Supplementary material for: The causal relationship between the human gut microbiota and pyogenic arthritis: a Mendelian randomization study
Source: Front Cell Infect Microbiol. 2024 Nov 26;14:1452480. doi: 10.3389/fcimb.2024.1452480 (PMC11629706; doi:10.3389/fcimb.2024.1452480)
Supplement: Supplementary file 1 [file DataSheet1.zip › mendelian test/ebi-a-GCST90027484.csv_forest.pdf]

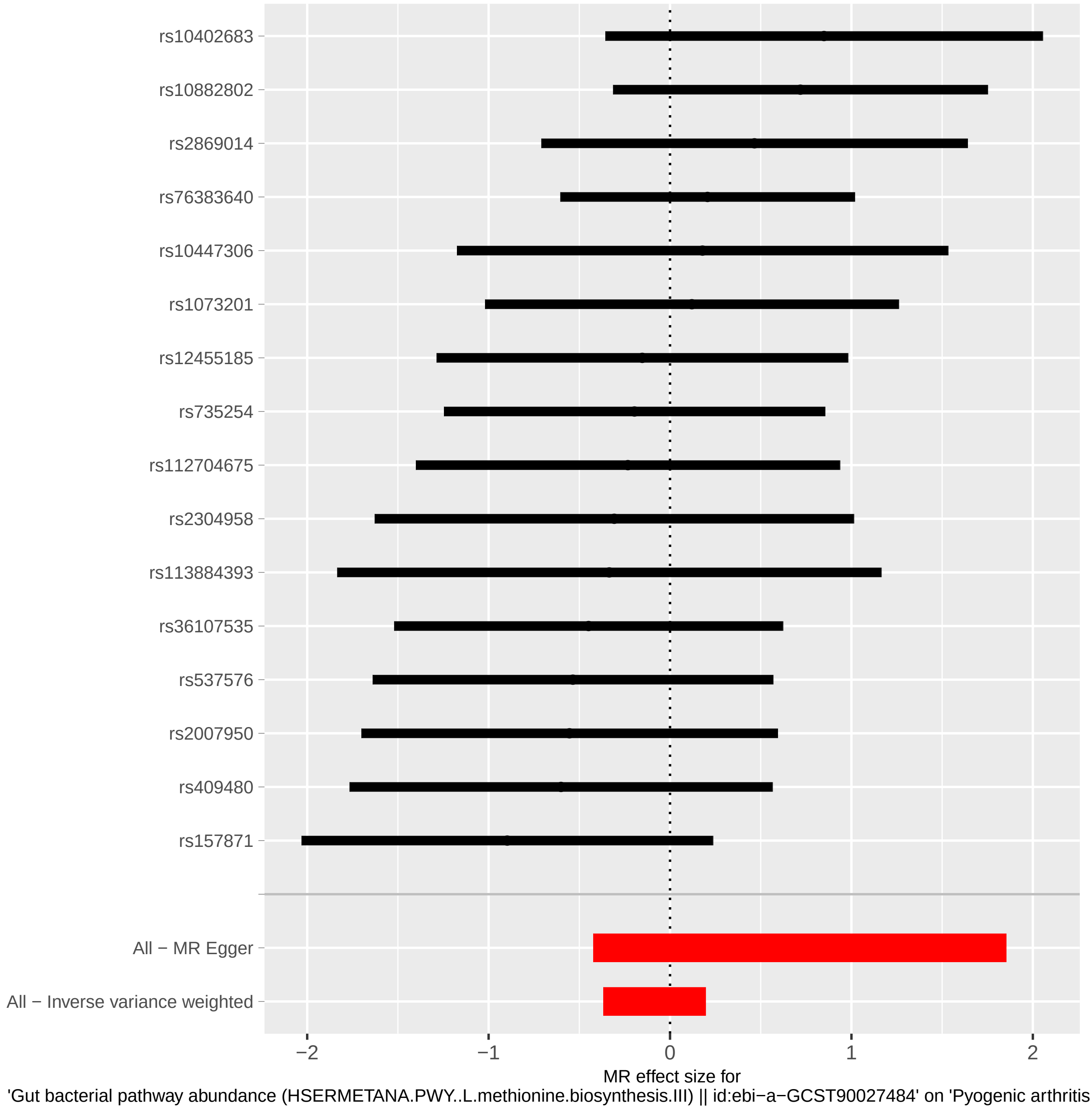

rs10402683
rs10882802
rs2869014
rs76383640
rs10447306
rs1073201
rs12455185
rs735254
rs112704675
rs2304958
rs113884393
rs36107535
rs537576
rs2007950
rs409480
rs157871
All − MR Egger
All − Inverse variance weighted
−2
−1
0
1
2
MR effect size for
'Gut bacterial pathway abundance (HSERMETANA.PWY..L.methionine.biosynthesis.III) || id:ebi−a−GCST90027484' on 'Pyogenic arthritis
